# Reporting of prognostic clinical prediction models based on machine learning methods in oncology needs to be improved

**DOI:** 10.1016/j.jclinepi.2021.06.024

**Published:** 2021-10

**Authors:** Paula Dhiman, Jie Ma, Constanza Andaur Navarro, Benjamin Speich, Garrett Bullock, Johanna AA Damen, Shona Kirtley, Lotty Hooft, Richard D Riley, Ben Van Calster, Karel G.M. Moons, Gary S. Collins

**Affiliations:** a Centre for Statistics in Medicine, Nuffield Department of Orthopaedics, Rheumatology and Musculoskeletal Sciences, University of Oxford, Oxford, OX3 7LD, UK; b NIHR Oxford Biomedical Research Centre, Oxford University Hospitals NHS Foundation Trust, Oxford, United Kingdom; c Julius Center for Health Sciences and Primary Care, University Medical Center Utrecht, Utrecht University, Utrecht, The Netherlands; d Department of Clinical Research, Basel Institute for Clinical Epidemiology and Biostatistics, University Hospital Basel, University of Basel, Basel, Switzerland; e Nuffield Department of Orthopaedics, Rheumatology, and Musculoskeletal Sciences, University of Oxford, Oxford, UK; f Centre for Prognosis Research, School of Medicine, Keele University, Staffordshire, UK. ST5 5BG; g Department of Development and Regeneration, KU Leuven, Leuven, Belgium.; h Department of Biomedical Data Sciences, Leiden University Medical Center, Leiden, the Netherlands.; i EPI-centre, KU Leuven, Leuven, Belgium

**Keywords:** Prediction, Machine learning, Reporting

## Abstract

**Objective:**

Evaluate the completeness of reporting of prognostic prediction models developed using machine learning methods in the field of oncology.

**Study design and setting:**

We conducted a systematic review, searching the MEDLINE and Embase databases between 01/01/2019 and 05/09/2019, for non-imaging studies developing a prognostic clinical prediction model using machine learning methods (as defined by primary study authors) in oncology. We used the Transparent Reporting of a multivariable prediction model for Individual Prognosis Or Diagnosis (TRIPOD) statement to assess the reporting quality of included publications. We described overall reporting adherence of included publications and by each section of TRIPOD.

**Results:**

Sixty-two publications met the inclusion criteria. 48 were development studies and 14 were development with validation studies. 152 models were developed across all publications. Median adherence to TRIPOD reporting items was 41% [range: 10%-67%] and at least 50% adherence was found in 19% (n=12/62) of publications. Adherence was lower in development only studies (median: 38% [range: 10%-67%]); and higher in development with validation studies (median: 49% [range: 33%-59%]).

**Conclusion:**

Reporting of clinical prediction models using machine learning in oncology is poor and needs urgent improvement, so readers and stakeholders can appraise the study methods, understand study findings, and reduce research waste.

**What is new?**

**Key findings**
• Reporting of prediction model studies in oncology based on machine learning methods is poor

**What this adds to what is known?**
• Specific areas for improvement include the reporting of title and abstract, study dates, sample size justification, missing data, description of flow and baseline characteristics of participants, performance measures (calibration and discrimination) and the presentation or availability of the prediction model

**What is the implication and what should change now?**
• Poor reporting is a barrier to the appraisal of study methods, understanding of study findings, and reducing research waste.
• Only when fully reported, can machine learning-based prediction models be evaluated by others; thereby increasing their chance of being used in clinical practice and without causing patient harm.
• Bespoke and robustly developed reporting guidance for prediction models based on machine learning methods is urgently needed. Till then, authors developing these models should use the TRIPOD reporting guideline to aid their reporting.

## 1 Introduction

Clinical prediction models are used extensively to aid medical decision making based on individual diagnosis, prognosis and risk [1], [2], [3], [4]. Oncology is a key area where prediction models are needed where they can help diagnose cancers, assess patient prognosis and guide patient treatment plans [4]. However, numerous studies have observed poor reporting of prediction models [5], [6], [7], particularly in the field of oncology [8], [9], [10], [11], [12], [13].

Incomplete reporting of essential information is a key barrier to the interpretation, further validation and uptake of clinical prediction models and contributes to the growing problem of research waste [14,15]. Poor reporting inhibits appraisal of applied study methods, understanding of study findings, prohibits independent validation by other researchers, and limits their inclusion in systematic reviews [16,17]. Consequently, this inhibits the eventual application and use in daily practice to facilitate clinical decision making.

To address poor reporting and improve the value of clinical prediction models, the Transparent Reporting of a multivariable prediction model for Individual Prognosis Or Diagnosis (TRIPOD) statement was published [18,19]. The TRIPOD Statement is a checklist of 22 items (comprising 37 sub-items) considered important to report in published reports describing the development and validation of a diagnostic or prognostic clinical prediction model with a focus on regression-based methods. Explicit guidance has also been developed for reporting Abstracts of prediction model studies [20].

Since the publication of the TRIPOD Statement, there has been a rapid and considerable interest to apply machine learning methods when developing a clinical prediction model [21]. Whilst there are numerous systematic reviews evaluating the methodological conduct and reporting of regression-based prediction models across a range of clinical areas, there is a dearth of research evaluating the completeness of reporting of machine learning based clinical prediction models [6,[22], [23], [24], [25]]. Though many essential reporting items overlap between regression-based and machine learning prediction modelling studies (e.g., study dates, sample size justification), we cannot assume that generally, reporting and methodological conduct between both types of models would be similar. Problematic areas for reporting are and how these differ for machine learning models compared to regression-based models is less known, and it is this area where machine learning models would occupy a different position to regression-based models and information is needed for the development of future guidelines. For example, analysis methods for machine learning differs, often more than one model is developed, and model availability/presentation is a barrier for many machine learning methods.

In this study, we evaluated the completeness of reporting of non-imaging prognostic prediction models developed using machine learning methods (as defined by the authors of the primary studies) in the field of oncology. Our findings will inform the development of the TRIPOD-AI reporting guideline [15].Methods

We systematically searched for non-imaging prognostic clinical prediction model studies using explicit machine learning methods (e.g., neural networks, random forests etc.) within the clinical area of oncology. Imaging and lab-based studies were excluded to ensure a review of prediction models developed in low dimensional, low signal and high noise settings and settings more reflective of the original TRIPOD statement.

As there is no universally agreed definition of machine learning (often viewed more as a difference in culture than methods [26]), we also included machine learning studies as defined by the authors of the primary reports. For example, studies using logistic regression were included if they were explicitly considered as machine learning by primary study authors, else it was excluded. We chose this definition to capture the reporting quality and style that authors of self-declared machine learning studies use and to avoid introducing another, possibly biased, dichotomy of what is or is not ML.

### 1.1 Protocol registration and reporting standards

We report this study according to the Preferred Reporting Items for Systematic Reviews and Meta-Analyses (PRISMA) guideline [27]. This study was registered with PROSPERO (ID: CRD42019140361) [28].

### 1.2 Information sources

We searched the MEDLINE and Embase medical literature databases via the OVID platform for studies developing oncology ML-CPMs published between 1 January 2019 and 5 September 2019. The search strategy included relevant MeSH or EMTREE subject headings and free-text terms, searched in the title, abstract or keyword fields, covering general modelling terms (such as “machine learning” or “statistical learning”), more specific ML modelling terms (such as “classification and regression tree”, “decision tree”, “random forest”, “naïve bayes”, “neural networks”, “support vector machine” “gradient boosting machine” and “K nearest neighbor”), cancer-related terms (such as “cancer”, “neoplasm” or “tumor”) and prediction terms (such as “predict”, “prognosis” or “risk”). Modelling, cancer and prediction search terms were then combined to retrieve publications satisfying all three sets of search terms. The search was limited to retrieve studies published in 2019 to ensure that a contemporary sample of studies were assessed in the review. No other search limits were applied. The search strategy was developed with an information specialist (SK). The full search strategies for both the MEDLINE and Embase databases are provided in Supplementary tables 1 and 2.

### 1.3 Eligibility criteria

Studies developing machine learning based prognostic prediction models in the field of oncology that were published in 2019, were included. Publications were eligible for this review based on the following inclusion and exclusion criteria:

#### 1.3.1 **Inclusion criteria:**

• Development of a prognostic prediction model:
  ○ using machine learning methods, as defined by authors
  ○ in the clinical area of oncology
  ○ for patient health related outcomes
  ○ for any outcome measurement (e.g., continuous, binary, ordinal, multinomial, time-to-event)
  ○ using at least two or more predictors in combination to produce an individualised patient predicted probability or classification
  ○ using any study design
    ■ experimental studies (including randomised controlled trials)
    ■ observational studies (including prospective studies, retrospective studies, cohort studies, case-control studies)
• English language studies

#### 1.3.2 **Exclusion criteria:**

• Studies with no reported development of a prediction model (validation only)
• Imaging studies, or studies using imaging parameters as candidate predictors
• Speech recognition/voice pattern studies, or studies using speech parameters as candidate predictors
• Lab-based studies
  ○ Genetic studies, or studies using genetic risk factors as candidate predictors
  ○ Molecular studies, or studies using molecular markers as candidate predictors
• Risk (prognostic) factor studies, primarily interested in the association of risk (prognostic) factors with the outcome
• Secondary research (e.g., reviews of prediction models)
• Conference abstracts

### 1.4 Study selection, data extraction and data management

Studies published during 2019 up until the final search date (September 5 2019) were selected to provide a contemporary sample of studies. Publications from MEDLINE and Embase were imported into Endnote reference software where they were de-duplicated and then imported into Rayyan web application where they were screened [29,30].

Two independent researchers (PD, JM) screened the titles and abstracts of the identified publications.

Two independent researchers, from a combination of five reviewers (PD, JM, GB, BS, CAN), reviewed the full text of potentially eligible publications and performed a double data extraction of eligible publications. One researcher (PD) screened and extracted data from all publications and four researchers (JM, GB, BS, CAN) collectively and independently screened and extracted data from the same articles. Disagreements were discussed and adjudicated by GSC, where necessary.

Data was extracted using a standardized data extraction form. The data extraction form was developed using a standardized and published TRIPOD adherence checklist and was piloted among all five reviewers using five eligible publications [31]. Results of the pilot were discussed, and TRIPOD adherence questions were clarified amongst all reviewers to ensure consistent data extraction. The data extraction form was amended by adding descriptive text to question for clarification, but text for questions was unchanged.

The data extraction form was implemented using Research Data Capture (REDCap) software [32].

### 1.5 Data items

Data items to be extracted from each publication were informed by the recommended items for measuring adherence to the TRIPOD reporting guideline [31]. Extracted data included reporting items from the title, abstract, introduction, methods, results, discussion, supplementary material and funding statements. The full list of TRIPOD adherence reporting items applicable for development only and development with validation studies are presented in Table 1. Descriptive data was also extracted on the cancer type, study design, type of prediction outcome, type of ML method used, intended use and if the aim of the clinical prediction model is to predict an individualized risk of value for each patient or classify patients in outcome groups (e.g., dead/alive).

**Table 1:** TRIPOD adherence reporting items

Reporting Items		Applicable to study type	Reporting items for TRIPOD adherence	
			Development only	Development with validation
**1. Title**		D,V	✓	✓
**2. Abstract**		D,V	✓	✓
**Introduction**				
**3. Background and objectives**	**a.** Context and rationale	D,V	✓	✓
	**b.** Objectives	D,V	✓	✓
**Methods**				
**4. Source of data**	**a.** Study design or source of data	D,V	✓	✓
	**b.** Key dates	D,V	✓	✓
**5. Participants**	**a.** Study setting (including number and location of centres)	D,V	✓	✓
	**b.** Eligibility criteria	D,V	✓	✓
	**c.** Details of treatment, if relevant	D,V	(✓)	(✓)
**6. Outcome**	**a.** Outcome definition (including how and when assessed)	D,V	✓	✓
	**b.** Blinding of outcome assessment	D,V	✓	✓
**7. Predictors**	**a.** Predictor definition (including how and when assessed)	D,V	✓	✓
	**b.** Blinding of predictor assessment	D,V	✓	✓
**8. Sample size**	Arrival at study size	D,V	✓	✓
**9. Missing Data**	Handling of missing data	D,V	✓	✓
**10. Statistical analysis**	**a.** Handling of predictors in the analysis	D	✓	✓
	**b.** Specification of the model, all model building procedures, and internal validation methods	D	✓	✓
	**c.** For validation, description of how predictions were made	V	✗	✓
	**d.** Specification of all measures used to assess model performance	D,V	✓	✓
	**e.** If done, description of model updating arising from validation	V	✗	(✓)
**11. Risk groups**	If done, details of how risk groups were created	D,V	(✓)	(✓)
**12. Development vs. validation**	For validation, description of differences between development and validation data	V	✗	✓
**Results**				
**13. Participants**	**a.** Description of flow of participants through the study	D,V	✓	✓
	**b.** Description of characteristics of participants	D,V	✓	✓
	**c.** For validation, comparison with development data	V	✗	✓
**14. Model development**	**a.** Number of participants and outcome in each analysis	D	✓	✓
	**b.** If done, unadjusted association between each candidate predictor and outcome	D	(✓)	(✓)
**15. Model specification**	**a.** Presentation of full prediction model	D	✓	✓
	**b.** Explanation of how to use the prediction model	D	✓	✓
**16. Model performance**	Report of model performance measures	D,V	✓	✓
**17. Model updating**	If done, report of results from any model updating	V	✗	(✓)
**Discussion**				
**18. Limitations**	Limitations	D,V	✓	✓
**19. Interpretation**	**a.** For validation, interpretation of performance measure results	V	✗	✓
	**b.** Overall interpretation of results	D,V	✓	✓
**20. Implications**	Potential clinical use of the model and implications for future research	D,V	✓	✓
**Other information**				
21. Supplementary*	Availability of supplementary resources	D,V	✗	✗
**22. Funding**	Source of funding and role of funders	D,V	✓	✓
**Total number of applicable items for TRIPOD adherence score**			**30**	**36**

D=development; V=validation

Parentheses () indicate conditional reporting items

⁎ 21. Supplementary is not used to calculate TRIPOD adherence score

The primary outcome of this systematic review is the adherence to the TRIPOD reporting guideline at the sub-item level (see Table 1) [31]. Risk of bias in individual studies and across studies was not assessed.

All data informing the analysis is available on the Open Science Framework (https://osf.io/2apwy/).

### 1.6 Summary measures and synthesis of results

Findings were summarized using descriptive statistics and visual plots, alongside a narrative synthesis. Analysis and synthesis of data was presented overall and by study type (i.e., development only studies and development studies with an external validation). Adherence to TRIPOD was calculated for each reporting (sub-) item and a TRIPOD adherence score was calculated for each publication, which equals the reporting for each developed model.

The supplementary material reporting item (item 21) was excluded from TRIPOD adherence score calculations for both study types. Reporting items specific to external validation were excluded from the TRIPOD adherence score calculation for development only studies (items 10c, 10e, 12, 13c, 17, and 19a). The total number of reporting items that were considered for the TRIPOD adherence score was a maximum of 30 for development only studies and 36 for development with validation studies.

The TRIPOD adherence score was calculated by dividing the total number of reported items by the total number of applicable reporting items for each respective study type. Three conditional reporting items applicable to the development of a model (‘Details of treatment, if relevant’ (item 5c), ‘If done, details of how risk groups were created’ (item 11) and ‘If done, unadjusted association between each candidate predictor and outcome’ (item 14b)) and two for the validation of a model (‘If done, description of model updating’ (item 10e) and ‘If done, report of results from any model updating’ (item 17)), were accounted for by reducing the total number of applicable reporting items (denominator) for each study type and publication accordingly. Therefore, the total number of reporting items that were considered for the TRIPOD adherence score was a minimum of 27 for development only studies and 31 for development with validation studies.

TRIPOD reporting adherence was compared between study type. All analyses were carried out in Stata v15 [33].

## 2 Results

**Fig. 1:**
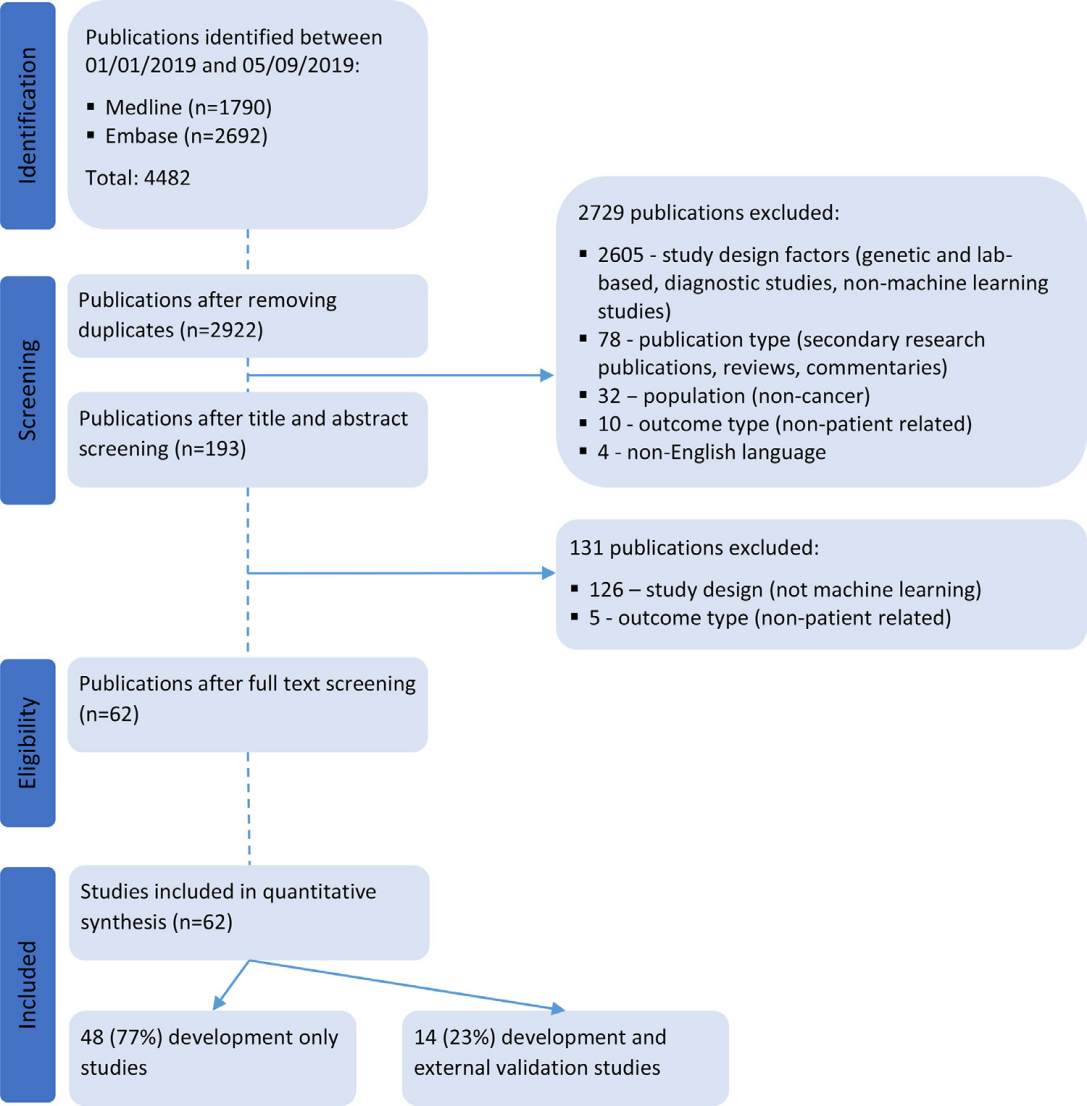
PRISMA flow diagram of studies included in the systematic review.

Our search strategy identified 2922 unique publications published between January 1 2019 and September 5 2019 indexed in the MEDLINE and Embase databases. 2860 publications were excluded during title and abstract screening and full text screening for not meeting the eligibility criteria. Reasons for exclusion were primarily study design, publication type and study population. We reviewed and extracted data from 62 publications (Fig. 1).

### 2.1 Study characteristics

Of the 62 publications, 48 (77%) were development only studies and 14 (23%) were development studies that also included an external validation of the developed model. Across the 62 included studies, 152 models were developed, with a median of two models reported per publication [range: 1-6]. There were 115 (76%) models from development only studies and 37 (24%) from the development with external validation studies.

Prediction models were developed primarily in lung cancer (*n*=8, 13%), breast cancer (*n*=6, 10%), colon cancer (*n*=6, 10%) and gynaecological cancers (*n*=6, 10%) (Table 2). Over half of the prediction models were for the intended use of healthcare providers (*n*=40, 65%). Most models were developed to predict binary outcomes (*n*=48, 77%) and 11 were predicting time-to-event outcomes (18%). Over half of the studies developed models with the aim to predict individualised risk (*n*=36, 58%), 25 with the aim to classify patients (41%) and one aimed to predict a value for the its continuous length of stay outcome. Six development only studies (13%) provided enough information to implement the model enabling predictions for new individuals, compared to four development with validation studies (29%). The most prevalent machine learning models were classification trees (n=28, 18%), logistic regression (*n*=27, 18%), random forest (*n*=23, 15%) and neural networks (n=18, 12%) (Table 3).

**Table 2:** Study characteristics of the included publications, by study type

Study characteristics	Development only (*n*=48)	Development and external validation (*n*=14)	All (*n*=62)
	*n*	*n*	*n*
**Cancer type**			
Lung	6	2	8
Breast	6	-	6
Colon/colorectal/rectal	3	3	6
Pancreatic	1	2	3
Liver	2	-	2
Gastric	3	-	3
Head and neck	5	-	5
Spinal	4	-	4
Brain (including meningioma, glioblastoma)	4	1	5
Oral (including nasopharyngeal carcinoma)	2	1	3
Gynaecological (including cervical, ovarian, endometrial)	5	1	6
Prostate/Penile	4	1	5
Skin (including melanoma)	1	1	2
Other*	2	2	4
**Outcome type**			
Binary	40	8	48
Continuous	-	1	1
Multinomial	2	-	2
Time to event	6	5	11
**Data source/study design^⁎⁎^**			
RCT	-	1	1
Prospective cohort	9	-	9
Retrospective cohort	11	3	14
Registry	15	6	21
Routine care database	7	2	9
Other**^⁎⁎⁎^**	2	1	3
Unclear	4	1	5
**Intended user**			
Health care providers	27	7	34
Public/patients	2	-	2
Researchers	1	-	1
Health care providers and patient/public	1	3	4
Health care providers and researchers	2	-	2
Unclear	15	4	19
**Aim of model**			
Predict risk	25	11	36
Predict length of stay (continuous outcome)	-	1	1
Classify patients	23	2	25
Sufficient information to apply the model and make predictions or classifications			
No	42	10	52
Yes	6	4	10

**Table 3:** Model characteristics of the included publications, by study type

Model characteristics	Development only (*n*=115 models)	Development and external validation (*n*=37 models)	All (*n*=152 models)
	*n* (%)	*n* (%)	*n* (%)
Regression-based models	30 (26)	12 (32)	42 (28)
Logistic regression	19	8	27
Cox regression	5	3	8
Linear regression	3	-	3
LASSO	2	1	3
Best subset regression	1	-	1
Alternative machine learning models	79 (69)	23 (62)	102 (67)
Neural network (including deep learning)	14	4	18
Random forest (including random survival forest)	19	4	23
Classification tree (e.g., CART decision tree)	25	3	28
Support vector machine	9	3	12
Gradient boosting machine	3	5	8
Naive Bayes	5	1	6
K nearest neighbours	1	2	3
Other^⁎⁎⁎⁎^	3	1	4
Ensemble models (n=8)	6 (5)	2 (5)	8 (5)
RUSBoost - boosted random forests	1	-	1
Bagging with J48 selected by Auto-WEKA	1	-	1
CoxBoost - boosted cox regression	1	-	1
XGBoost: exTreme Gradient Boosting	-	1	1
Gradient boosting machine and Nystroem, combined using elastic net	-	1	1
Adaboost	1	-	1
Bagging, method not specified	1	-	1
Partitioning Around Medoid algorithm and complete linkage method	1	-	1
Median number of models developed per study [IQR], range	2 [1 to 4], 1 to 6	2 [1 to 5], 1 to 6	2 [1 to 4], 1 to 6

*Other includes peritoneal carcinomatosis, incurable cancer (various), leukemia, malignant peripheral nerve sheath tumour

**validation characteristics for data source/study design are: RCT: 2/14 (14%); prospective cohort: 3/14 (21%); retrospective cohort: 4/14 (29%); registry: 2/14 (14%); routine care database: 2/14 (14%); Other: 1/14 (7%).

***Other includes a combination of data sources (Indian hospitals, SEER research database and data from research centres), a survey and an audit.

****Other includes voted perceptron; fuzzy logic, soft set theory and soft set computing; hierarchical clustering model based on the unsupervised learning for survival data using the distance matrix of survival curves; Bayes point machine

### 2.2 TRIPOD Adherence

Overall, publications adhered to between 10% and 67% of the TRIPOD reporting items and had a median adherence of 41% (Table 4). Development only studies showed poorer reporting adherence to TRIPOD (median: 38% [range: 10%-67%]) compared to development with validation studies (median: 49% [range: 33%-59%]). Scoring for each included publication is provided in Supplementary table 3.

**Table 4:** Median and range of reporting adherence to TRIPOD

	TRIPOD Adherence Score (%)		
	*n*	Median [IQR]	Range (%)
Overall	62	41 [34 to 48]	10 to 67
Study type			
*Development only*	48	38 [34 to 45]	10 to 67
*Development with validation*	14	49 [38 to 56]	33 to 59
Number of models developed in study			
*1*	26	41 [34 to 50]	17 to 67
*2*	13	38 [36 to 45]	31 to 59
*3*	6	34 [17 to 38]	10 to 45
*4*	6	41 [38 to 41]	31 to 52
*5*	8	41 [31 to 48]	17 to 59
*6*	3	47 [14 to 55]	14 to 55

**Fig. 2:**
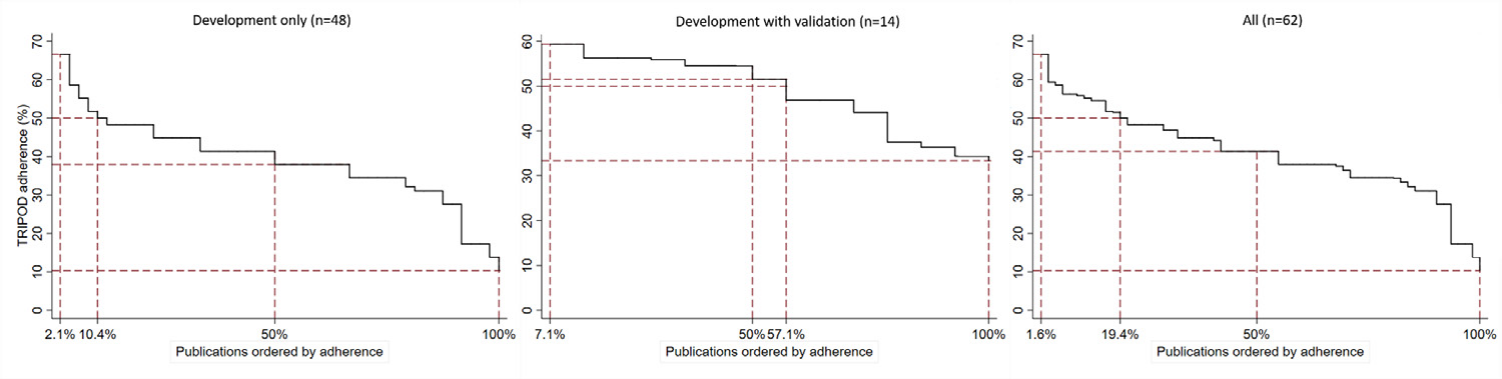
Reporting adherence to TRIPOD across publications, overall and by study type.

Figure 2 summarises the reporting adherence across publications. At least 50% TRIPOD adherence was achieved by 19% of publications overall and 10% and 57% of development only and development with validation studies, respectively.

**Fig. 3:**
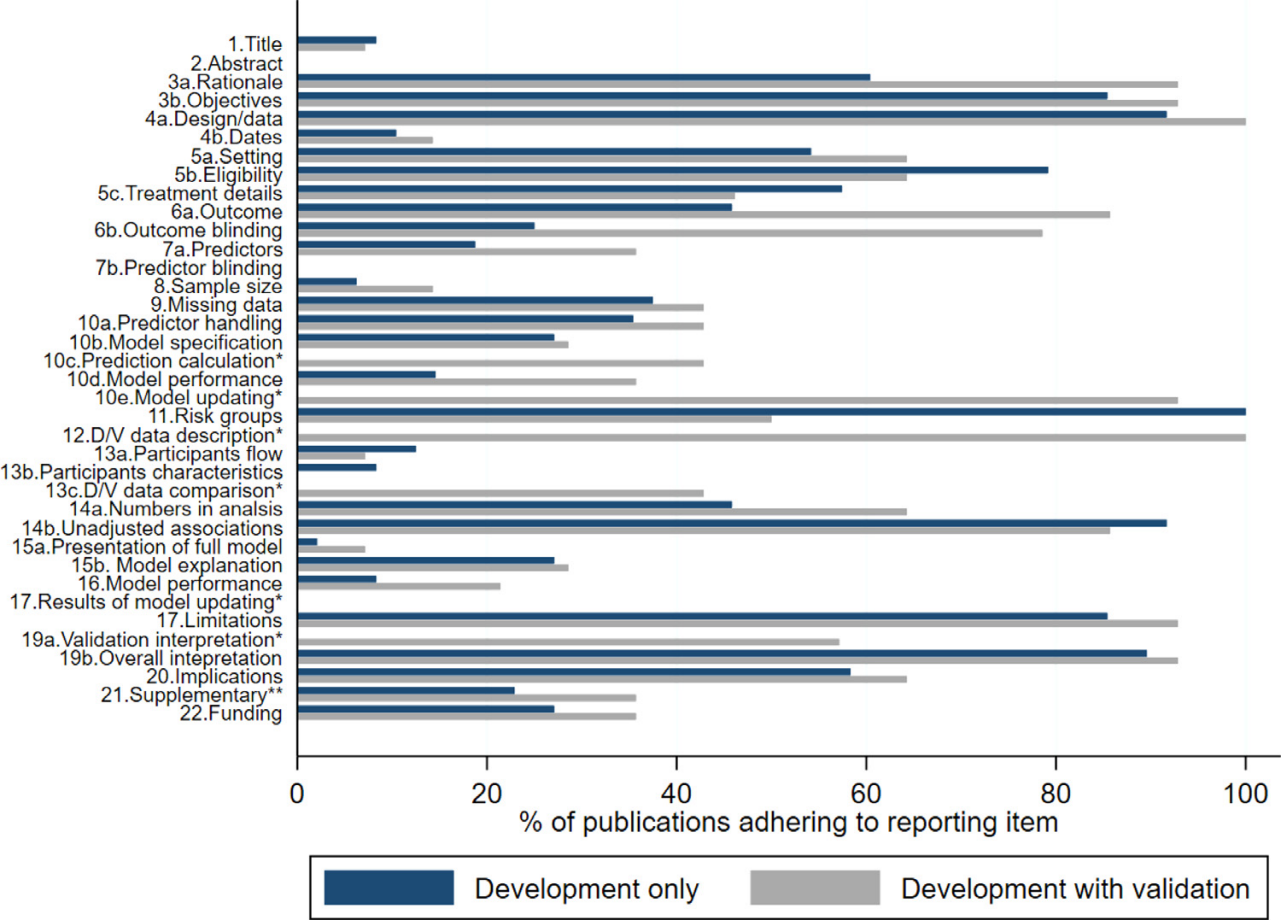
Reporting of the items of the TRIPOD statement, for development only (*n*=48) and development with validation studies (*n*=14). See Table 1 for the full list of TRIPOD reporting items. *item not applicable for development study; **item not included in scoring. D/V=Development/validation.

Figure 3 summarises the completeness of items for each section of the TRIPOD statement (Title, Abstract, Introduction, Methods, Results, Discussion and Other information), by study type, for which we describe key findings below. A complete breakdown of reporting is provided in Supplementary tables 4.

#### 2.2.1 Title and abstract (items 1 and 2)

The TRIPOD statement asks that prediction modelling studies identify the prediction element and the study type (development and/or validation of a prediction model) in the title, with the target population and outcome to be predicted. Four development only studies and one development with validation study fully reported these four elements in the title of their publication. Study type was the most poorly reported with 21% (*n*=10) of development studies reporting their study type and no development with validation studies.

Key information for the abstract includes a summary of objectives, study design, setting, participants, sample size, predictors, outcome, statistical analysis, results, and conclusions. Neither the development nor development with validation studies reported the abstract fully at all in their publications. Study objectives were poorly reported for development with validation studies (n=4, 29%) compared to development only studies (*n*=41, 85%). Study conclusions were well reported overall (*n*=57, 92%).

#### 2.2.2 Introduction (item 3)

Background and objectives include study details on the context and rationale (item 3a) and objectives (item 3b) of the study. Study objectives were well reported for both study designs. Reporting of the context and rationale was 60% (*n*=29) for development studies but was higher for development with validation studies (*n*=13, 93%).

#### 2.2.3 Methods (items 4-12)

Reporting of the methods section was variable for both study designs, but overall better reporting was observed in development with validation studies. Study design and the source of data was well reported for all publications (*n*=58, 93.6%) but key study dates (start and end of accrual, length of follow-up and prediction horizon) were poorly reported (*n*=7, 11.3%). Eligibility of participants was well reported in both study design publications (*n*=47, 76%) but 56% (*n*=35) and 53% (*n*=33) of studies reported the study setting and details of treatment, respectively (if applicable).

Outcome definition and blinding of outcome assessment was better reported in development with validation studies (*n*=12, 86%; *n*=11, 79%, respectively) than development studies (*n*=22, 46%; *n*=12, 25% respectively). Similar results were found for predictor definitions, though blinding of predictor assessments was not explicitly reported in any publication. Sample size justification was reported in five publications (8%) and 39% (*n*=24) reported how missing data was handled (including reporting of methods to handle missing data, software used, and details of imputation). Details on how risk groups were created was reported for four out of five applicable studies.

Reporting the analysis was poor; two-thirds of development only studies failed to report how predictors were handled in the modelling (*n*=31), three-quarters did not fully describe the type of model (i.e., did not report all model building and internal validation steps) (*n*=35) and 85% did not specify the model performance measures in the Methods section. Reporting of these items was better in development with validation studies, in which 93% (*n*=13/14) of studies also reported a description of how the models were updating, if it was done. Half of the development with validation studies provided a description of differences between the development and validation datasets.

#### 2.2.4 Results (items 13-17)

Reporting of results was poor and variable. 89% of publications did not describe the flow of participants in their study, including the number of participants with and without the outcome and, if applicable, a summary of the follow-up time. 94% did not fully describe participant characteristics (basic demographics, clinical features, available predictors), including the number of participants with missing data for predictors and outcome. Six (43%) development with validation studies compared development and validation datasets. Reporting of unadjusted association between candidate predictor and the outcome, however, was very high (*n*=56, 90%).

Presentation of the full (final) model was provided in two studies (3%) for four logistic regression models. One (2%) study provided code for its deep learning-based survival analysis and five studies (8%) provided reference to a web calculator. Explanation of how to use the prediction model was infrequent (*n*=17, 27%). Four out of five applicable studies reported the boundaries of created risk groups.

Discrimination was reported in 76% (*n*=47/62) of studies and calibration was reported in 18% (*n*=11/62). Discrimination measures were predominantly the area under the receiver operating characteristic curve (AUC and c-statistic) (*n*=38/47, 8%), or measures analogous to this (c-index) (*n*=8/47, 17%). Calibration measures included the calibration plot (*n*=9/11, 82%) and one Hosmer-Lemeshow test. One study reported the root mean square log error and presented a plot of average actual length of stay for each tenth and average predicted values when predicting continuous length of stay. Only 7 studies (11%) adequately reported both discrimination and calibration model performance measures (including confidence intervals). The proportion of studies reporting discrimination and calibration was higher in development with validation studies (*n*=8, 57% and *n*=6, 43% respectively) compared to development only studies (*n*=17, 35% and *n*=9, 19% respectively).

#### 2.2.5 Discussion (items 18-20) and other information (items 21 and 22)

Better overall reporting was found in the discussion. Study limitations and overall interpretation of the results were well reported (*n*=54, 87%; *n*=56, 90%, respectively). 57% of development with validation studies reported an interpretation of validation performance measures and 60% of all studies reported potential clinical use of the model. Funding (source and role) was fully reported in 18 studies (29%).

## 3 Discussion

### 3.1 Summary of findings

In this systematic review, we assessed the quality of the reporting of studies describing the development (including validation) of author defined machine learning prediction models in the clinical field of oncology. Inadequate reporting of essential reporting items for prediction modelling was found in all included publications. Though reporting was better in development with validation studies (compared to development only studies), most publications reported less than half of the essential information needed when developing and validating a prediction model. Reporting of items that were specific to the validation of the prediction model was better than the development aspect.

Reporting was very poor in all sections of the published reports except in the Discussion, where authors reported the overall interpretation of the results and limitations of the study well. However, the full model (or link to code or a web calculator) was rarely provided despite logistic regression (labelled as machine learning) being a prevalent machine learning method used to develop the models, which can be presented in full, and many online platforms to make the model code available. The recommended performance measures were also rarely provided (e.g., calibration and discrimination), and thus would be inadequate evidence to support the overall interpretation given in the discussion.

The title and abstract, which are made up from multiple reporting sub-items, failed to be fully compliant with the title and abstract reporting recommendations in TRIPOD in most studies, respectively. This can particularly affect the findability and usability of research where studies may not be retrieved by literatures searches or be indexed appropriately in databases and is another factor to the lack of evaluation of models and thus lack of use in clinical practice. The methods and results sections followed in a similar suit where reporting was more variable. Particularly problematic areas were reporting on key study dates, predictor assessment blinding, justification of the sample size, participant flow and description of baseline characteristics and presentation of the full model.

With the rapid growth and interest in applying machine learning methods for predicting health outcomes for individual patients, it is of paramount importance that all necessary information needed for reproducibility and to critically appraise the study methods is fully reported, enabling readers to identify biases and judge and interpret the study findings. In the absence of key information, e.g., if the target population for whom the model is intended is not clearly described, or the intended moment to use the model has not been reported then using such models can potentially cause harm. If a model has not been fully reported or a link to the availability of the model not given, then implementation or evaluation (by independent investigators) is hampered or not possible. For validation studies, studies which fail to report key details including performance measures (discrimination and calibration) then synthesising these results in a systematic review is challenging, potentially excluding studies. We have recently seen these harmful implications from poor reporting in the living systematic review of COVID-19 prediction models where most models were found to be poorly reported making them unusable [7].

### 3.2 Literature

There is limited evidence on the reporting quality of full text publications for studies developing or validating machine learning and regression-based prediction models in oncology.

A systematic review that assessed reporting quality based on TRIPOD of abstracts of publications from oncology journals similarly found issues in the methodological aspects of reporting in their included clinical prediction model studies, for example, study design, settings and statistics [34]. However, this study has only been published as a supplement and is currently ongoing, and full details are not available - it is unclear if the sample of papers included models developed using machine learning. An assessment of full text reporting quality has not yet been conducted in the clinical area of oncology and machine learning based prediction models.

Heus et al formally assessed reporting quality and adherence to TRIPOD of full text publications for studies developing or validating regression-based multivariable prediction model studies, in which oncology was one of 37 clinical domains studied [35]. Studies published before the introduction of the TRIPOD statement in 2015 and non-regression-based techniques, such as machine learning, were excluded. The review also included validation only and diagnostic studies. However, the findings from Heus et al were comparable with findings from our review, though we found a lower TRIPOD adherence score in our review.

Heus et al found similar reporting items particularly problematic for regression based prediction models that we found for machine learning based models, such as title, abstract, actions for blinding, description of participants characteristics, predictive accuracy of the models and presentation of the full model. Better reporting was also observed for regression-based prediction models in the source and study design in the methods, risk groups and items in the discussion.

However, we observed that reporting of objectives was better, and context and rationale was worse, reporting of key study dates was worse and reporting of unadjusted associations (if done) was better for studies developing machine learning based prediction models. We observed that in addition to describing participants characteristics, describing the participant flow into the study was poor; and in addition to reporting blinding of predictor assessment, reporting predictor definitions was poor. Reporting of how the sample size was arrived at was also poor in our review.

Results from several systematic reviews informally assessing reporting quality and TRIPOD adherence, in oncology and otherwise, are comparable with our findings [6,8,22,24,25,[36], [37], [38]]. A systematic review of regression-based models found inappropriate methods and poor reporting when developing models in cancer [8] and review of machine learning methods using routinely collected data in intensive care identified poor methodological and reporting as barriers to the use of prediction models in clinical practice [37]. A pre- and post- review of prediction models found that though reporting has improved since TRIPOD have been published, reporting issues remain [38].

### 3.3 Strength and limitations

We add to a building body of evidence that has found poor and variable reporting of prediction models, irrespective of modelling methods. We shed light on not only reporting practice and quality in oncology prediction model studies, but also on the rapidly growing use of machine learning.

We used the recommended TRIPOD reporting statement for prediction modelling, which was designed for regression-based prediction models, to assess the quality of reporting of models developed using machine learning. Though some items of this reporting statement may be harder to adhere to (e.g., presentation of the prediction model), nearly all items in the TRIPOD Statement are applicable for machine learning based prediction model studies given its emphasis placed on reporting items for overall study design and the non-specific terminology used for methods, modelling approaches and performance measure reporting items. By selecting papers where authors declare using machine learning, we select papers where authors may not think TRIPOD is applicable, however, other machine learning reporting guidance is available, such as Minimum information about clinical artificial intelligence modelling (MI-CLAIM) checklist and reporting guideline by Luo et al [39,40]. It is unlikely these guidelines would alter the findings and conclusions of this study as there is overlap in essential reporting items with TRIPOD such as study design, rationale, methods and performance measures.

Though we used two major information databases to search for studies developing machine learning based prediction models in oncology (MEDLINE and Embase), it is possible that some eligible publications may have been missed. Further, given the speed of publication in this field many models will have been published since the search dates of this study. However, we selected a contemporary sample of publications from 2019 and given comparability of our findings to the current literature, it is unlikely that additional studies would change the conclusion of this review.

### 3.4 Future research

In response to the increased development of machine learning based prediction models and the challenges associated with reporting machine learning clinical prediction models, including conflicting terminology, the TRIPOD collaboration has initiated the development of a TRIPOD Statement specific to machine learning (TRIPOD-AI) [15]. This will raise awareness of essential reporting items for prediction modelling using machine learning and help standardise reporting [15]. Periodic reviews and re-reviews of prediction modes are warranted in oncology, and other clinical domains, to continue to evaluate completeness reporting as they change in this quick evolving field of research. Fuller reporting for essential items in clinical prediction model studies can also be aided by publishing journals by endorsing reporting guidelines and allowing space for more detail in publications.

## 4 Conclusions

Reporting of machine learning-based prediction models in oncology is poor and needs to be improved to allow for further validations and increasing their chance of being used in clinical practice and without causing patient harm. Specific areas for which reporting needs to be improved include the title and abstract, sample size justification, description of flow and baseline characteristics of participants and the presentation of the full models.

## Author contributions

PD and GSC conceived the study. PD, CAN, BVC, KGMM and GSC designed the study. PD and SK developed the search strategy. PD and JM carried out the screening. PD, JM, CAN, BS, and GB carried out the data extraction of all items from all articles. PD performed the analysis and drafted the first draft. PD, JM, CAN, BS, GB, JAAD, SK, LH, RDR, BVC, KGMM, and GSC critically reviewed and edited the article. All authors read and approved the final manuscript.

## Acknowledgements

None

## Appendix Supplementary materials

Image, application 1
